# Association Between Perceived Stress and Salivary Biomarkers of Allostatic Load Among Gender Minorities in Chennai: An Observational Cross-Sectional Study

**DOI:** 10.7759/cureus.46065

**Published:** 2023-09-27

**Authors:** Mahalakshmi Kumaraguru, Lalitha Rani Chellappa, Meignana Arumugham I, Selvaraj Jayaraman

**Affiliations:** 1 Department of Public Health Dentistry, Saveetha Dental College & Hospital, Saveetha Institute of Medical and Technical Sciences, Saveetha University, Chennai, IND; 2 Department of Biochemistry, Saveetha Dental College & Hospital, Saveetha Institute of Medical and Technical Sciences, Saveetha University, Chennai, IND

**Keywords:** gender nonconforming, biomarkers, saliva, stress, transgender

## Abstract

Background

Transgender individuals in India experience immense psychosocial stressors, stigma, and violence. In response to stress, the body exhibits adaptive responses that necessitate the production of organic chemicals ensuing in the detection of blood serum and saliva. There are currently no laboratory tests that are confirmatory for the diagnosis of stress and facilitate necessary treatment to be carried out in a timely manner. Thus, potential salivary biomarkers could be a helpful tool in overseeing the efficacy of pharmacological treatment prescribed by a psychiatrist.

Aim

This study aimed to assess the correlation between perceived stress and salivary stress biomarker levels in transgender and gender nonconforming (TGNC) individuals in Chennai, India.

Methodology

Twenty-two TGNC individuals and 22 age-matched controls in Chennai were administered the Perceived Stress Scale-10 questionnaire. Following this, their saliva samples were collected using the passive drool technique and subjected to sandwich enzyme-linked immunosorbent assay (ELISA) technique for measuring salivary cortisol, salivary tumor necrosis factor-alpha (TNF-alpha), and salivary C-reactive protein (CRP). Independent t-test was used to compare salivary stress biomarker levels between the TGNC and age-matched control groups. Pearson’s correlation test was done to correlate perceived stress and salivary stress biomarker levels in the TGNC group.

Results

Significant difference was seen between the TGNC and control groups with respect to salivary cortisol and salivary TNF-alpha levels, with the levels being higher in the TGNC group. A significant positive correlation was seen between perceived stress and salivary cortisol and between perceived stress and salivary TNF-alpha levels.

Conclusion

There is a significant correlation between perceived stress and salivary biomarkers of stress among TGNC people in Chennai.

## Introduction

Transgender persons make up an estimated 25 million people or 0.3-0.5% of the world's population. In India, 31% of Hijras die by suicide, and 50% of them attempt it at least once before turning 20 [1]. These figures make up the quintessence of this study. Having a gender identification that does not correspond with one's assigned sex at birth is known as being transgender or gender nonconforming (TGNC), which is a very broad notion [2]. The information on transgender people in India was gathered in the 2011 census under the category "others," which also includes anyone who wants to record sex information in addition to transgender people. A total of 4,87,803 people are classified as "others" according to the 2011 census [3]. Despite being recognized in the country's census, many transgender and gender-variant people in India struggle to access essential healthcare services owing to the social stigma associated with their gender identity. To understand how this stigma impacts TGNC people in different contexts, the minority stress theory was first introduced by Ilan Meyer, which describes the disparities in psychological health among sexual minorities [4].

Such disparities in psychological health stem from a variety of problems and interpersonal difficulties, which include desertion from kin and peers, poor self-regard, and negative body image resulting from an effort to reject the body parts/bodies they do not identify with [5].

According to Hembree et al. [6], gender incongruence may cause distress that is known clinically as gender dysphoria (GD). Through its influence on behavioral, metabolic, and hormonal pathways, psychosocial stress connected to the encounter with social stigma or discrimination has been shown to operate as a contributory factor to health inequalities [7].

Allostasis describes the body's active oscillation in reaction to stressful changing circumstances, as opposed to homeostasis, where the internal environment is constant [8]. The hypothalamic-pituitary-adrenal (HPA) axis and the sympathetic-adrenomedullary axis are triggered in reciprocation to oscillation, ultimately stimulating a chain of physiological reactions that include the release of primary mediators, such as neuroendocrine chemical messengers, like cortisol, epinephrine, and norepinephrine, and immune and inflammatory chemical messengers, like interleukin-6 (IL-6) and tumor necrosis factor-α (TNF-α) that facilitate the body to withstand stress [9]. Eventually, a secondary physiologic reaction is incited in response to the primary mediators, which could vary from cardiovascular disorders, such as hypertension and irregular pulse, to metabolic disorders (elevated waist-hip ratio, dyslipidemia, and elevated glucose levels) and inflammatory reactions, such as the release of fibrinogen and C-reactive protein (CRP) [10]. The final output of the above-mentioned events is the establishment of an allostatic load (AL) model, which represents the cumulative effect of the stress undergone on the mental and physical well-being of the body in a particular time frame, thus painting a comprehensive image of the body’s adaptation mechanism to excessive stress [8].

Although mental health issues may be self-limiting, delaying or avoiding formal medical healthcare care can lead to deleterious consequences. Moreover, the longer the duration of persisting poor mental health, the worse the psychological consequences, such as depression. Hence, salivary biomarkers can prove to be a quick and potential means for the diagnosis and treatment of stress and anxiety, if its correlation with mental health can be established beyond doubt. Moreover, the specific health needs of transgender individuals must be identified as a priority research area due to the population’s unique health experiences. Hence, this study aimed to correlate the perceived stress levels among TGNC people with salivary biomarkers of stress. To the best of our knowledge, no previous research has studied the interactions between these factors.

## Materials and methods

Study design and setting

An observational cross-sectional study was carried out among TGNC individuals in Chennai in the month of April 2022.

Ethical clearance

Ethical clearance was obtained from the scientific review board of Saveetha Dental College and Hospital (approval no. SRB/SDC/PHD-2103/22/029).

Study population

TGNC individuals as the test group and gender majority individuals attending the outpatient department of Saveetha Dental College and Hospital as the control group constituted the study population.

Inclusion criteria and exclusion criteria

Self-identified TGNC individuals, who declared their willingness to take part in the study, aged between 18 and 65 years, were eligible to be included in the study. Intellectually disabled individuals, individuals undergoing hormonal treatment, and participants with HIV/hepatitis and other immunocompromised systemic conditions were excluded from the study. Participation in the study was voluntary (all participants signed a written informed consent) and anonymous.

Sample size calculation

The sample size was calculated based on a previous study done by Arvind et al. [11] using G*Power software version 3.1.9.7. The final sample size was 44, which included 22 TGNC individuals and 22 controls (gender majority). Although males and females perceive stress differently, they both belong to the gender majority and hence significantly vary from the way gender minorities perceive stress and were thus grouped together to be a part of the control group.

Sampling and data collection

As the TGNC population is a hard-to-reach and highly marginalized community, they were recruited for the study using a snowball sampling technique through the inclusion and exclusion criteria until the required sample size was reached. Age-matched controls were recruited from the outpatient department of Saveetha Dental College and Hospital in Chennai, India, using a simple random sampling method.

Saliva collection and analysis

All saliva collection sessions were meticulously scheduled during the afternoon in order to reduce the effect of diurnal variation, thus ensuring a more precise and uniform evaluation of biomarkers. Thirty minutes before the collection, the participants were instructed to withhold from performing oral hygiene and refrain from consuming alcohol, tobacco, caffeine, and dairy products. Stress biomarkers were measured before the survey. Pre-trained researchers who analyzed the saliva samples were blinded to the sample source to reduce possible bias.

Unstimulated saliva was collected using the passive-drool technique. In less than an hour from the time of saliva collection, the samples were transferred for storage at a temperature of −70 °C until the samples were subjected to biochemical analysis. Only the clear supernatant part of the sample was subjected to analysis after centrifugation of samples at 3000 rpm for 15 minutes.

Salivary cortisol levels were assessed with the Abbkine Human Cortisol (COR) ELISA Kit (Abbkine Scientific Co., Ltd, USA) with a calibration range of 12.5-200 μg/L and a 1.0 μg/L detection limit. The kit facilitates the use of a two-site sandwich ELISA technique to measure cortisol in samples. The kit contains a microplate with a coating of a cortisol-specific antibody. Standards and samples are pipetted into the wells, and any cortisol present is bound by the immobilized antibody. Horseradish peroxidase (HRP)-conjugate human cortisol detection antibody is added to the wells following the elimination of any unbound substances. The well is washed to eliminate any unbound HRP reagent, after which a chromogen solution is added to the wells to result in the development of a color, the intensity of which is proportional to the quantity of cortisol bound in the first step. The color development is then terminated, and the intensity of color is quantified and measured. 

Similar procedures employing sandwich ELISA were done using EliKine™ Mouse TNF-α ELISA Kit (Abbkine Scientific Co., Ltd, USA) (calibration range: 15.6 pg/ml-1000 pg/ml, limit of detection: 8 pg/mL) and Rat CRP ELISA Kit (Abbkine Scientific Co., Ltd, USA) (calibration range: 150 μg/L-2400 μg/L, limit of detection: 15 μg/L).

The same saliva collection and analytical methods were followed for healthy age-matched controls who reported to the outpatient department of Saveetha Dental College and Hospital.

Questionnaire

The 10-item Perceived Stress Scale (PSS-10) questionnaire was administered by the principal investigator prior to beginning the saliva sample collection [12]. The questionnaire was self-administered, but it was explained to the participant in English and the regional language (Tamil). This is adapted from the original 14-item PSS self-report measure [13] designed to assess the degree to which situations in one’s life are appraised as stressful.

The items are rated on a five-point frequency scale, ranging from "never" (0) to "very often" (4) in the last month. To calculate the final PSS score for an individual the responses for the four positively stated items (4, 5, 7, and 8) were reversed (e.g., 0 = 4, 1 = 3, 2 = 2, 3 = 1, and 4 = 0), followed by the addition of all the scale items. Individual scores on the PSS can range from 0 to 40, with greater scores representative of higher perceived stress. The PSS scores were categorized as low (0-13), moderate (14-26), and high (27-40).

Statistical analysis

The data were entered in an Excel sheet and analyzed using IBM SPSS Statistics for Windows, version 23 (released 2015; IBM Corp., Armonk, New York, United States). Descriptive statistics were carried out to determine the frequency and distribution of the control and TGNC groups according to stress levels. The difference in mean salivary biomarker levels between the TGNC group and control group was analyzed using independent t-test. Pearson’s correlation test was employed to correlate perceived stress and salivary stress biomarker levels in TGNC people.

## Results

**Figure 1:**
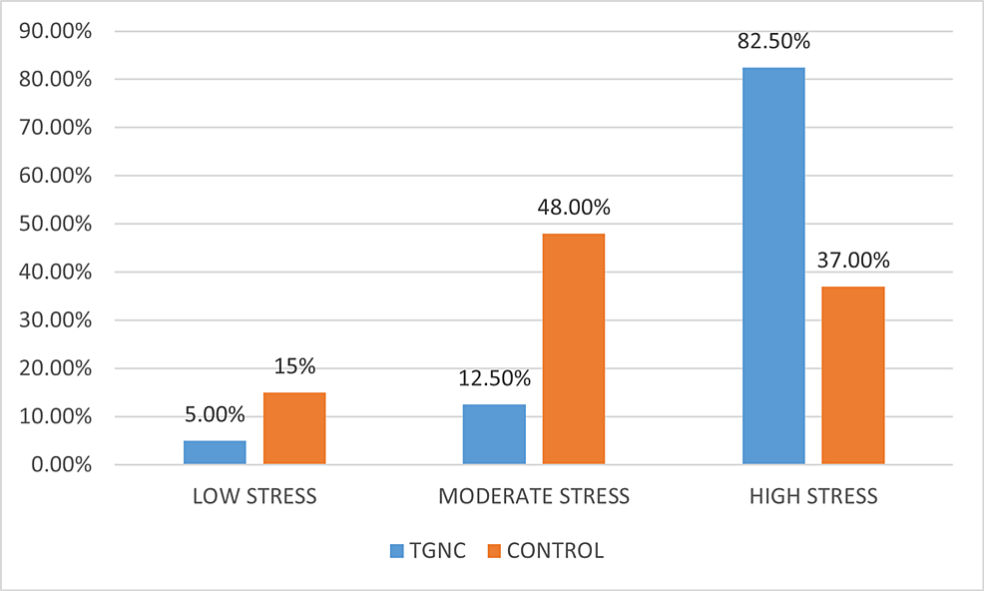
Distribution of the controls and TGNC subjects according to the perceived stress levels TGNC: transgender and gender nonconforming

**Figure 2:**
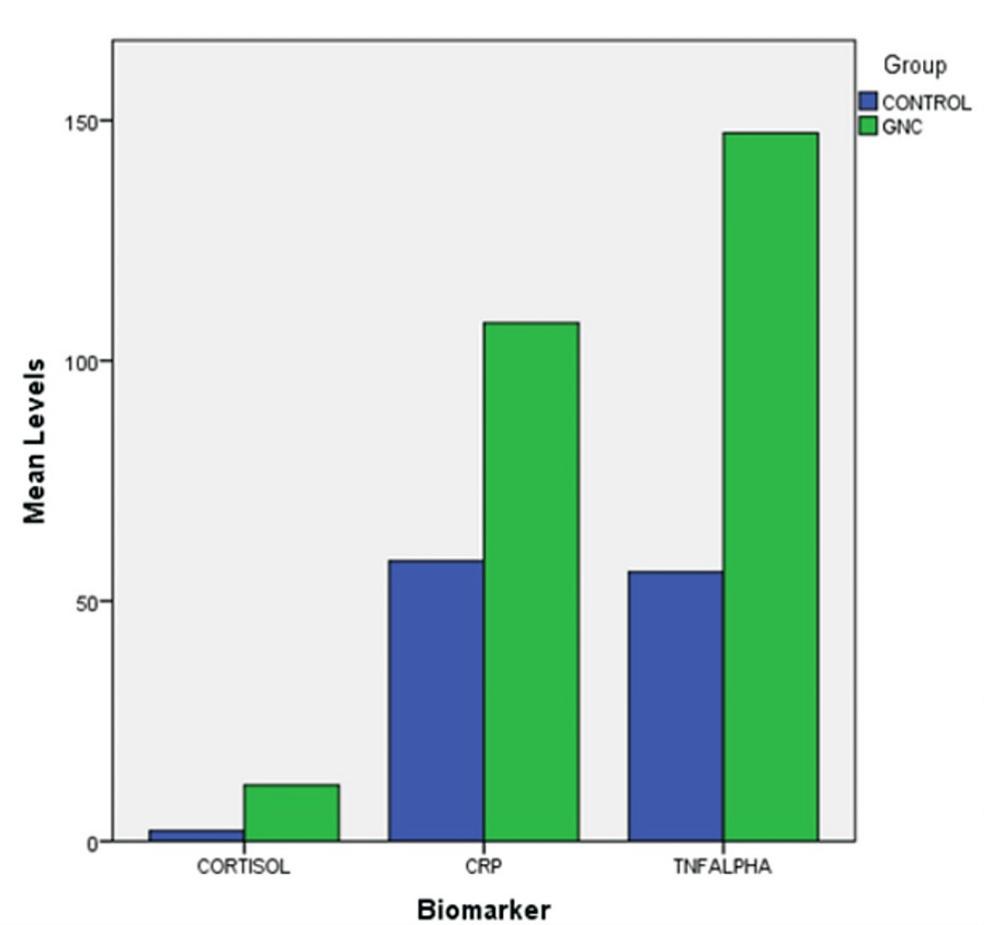
Mean salivary biomarker levels in the control and TGNC groups GNC: gender nonconforming; TGNC: transgender and gender nonconforming; CRP: C-reactive protein; TNFALPHA: tumor necrosis factor alpha

The mean PSS-10 score of the TGNC individuals was 30.9+/-6.725, and the mean PSS-10 score among the controls was 18.75+/-5.644. Figure 1 shows the percentage distribution and frequency of the controls and TGNC individuals according to their PSS-10 categorization, where in the TGNC group, 63.6% (n=14) had high perceived stress, 12.5% (n=5) had moderate perceived stress, and only 5% (n=2) of the TGNC group had low-stress levels. Table 1 demonstrates the mean salivary stress biomarker levels of both the control and TGNC groups, as illustrated in Figure 2, in which the mean cortisol, mean TNF-α, and mean CRP levels in the saliva of the control group were 2.152, 56.019, and 58.319 nmol/L, respectively. Meanwhile, the mean cortisol, mean TNF-α, and mean CRP levels in the saliva of the TGNC group were 11.668, 147.396, and 107.841 nmol/L, respectively. A statistically significant difference was present between the control and TGNC groups concerning salivary cortisol and salivary TNF-α levels, but no significant difference was found concerning the CRP levels. Table 2 illustrates the correlation between the PSS-10 scores and salivary stress biomarker levels in the TGNC individuals, demonstrating a significant correlation between perceived stress and salivary cortisol levels, as well as perceived stress and salivary TNF-α levels. However, no significant correlation was obtained between perceived stress and salivary CRP levels.

**Table 1:** Comparison of salivary stress biomarker levels between the TGNC and control groups using independent T-test TGNC: transgender and gender nonconforming; TNF: tumur necrosis factor; CRP: C-reactive protein

T-test	Mean (nmol/L)	Standard deviation	P value
Control cortisol	2.152	0.698	0.000
TGNC cortisol	11.668	11.414	
Control TNF-alpha	56.019	21.384	0.000
TGNC TNF-alpha	147.396	69.927	
Control CRP	58.319	11.710	0.84
TGNC CRP	107.841	14.165	

**Table 2:** Correlation between perceived stress and salivary stress biomarker levels in the TGNC group using Pearson’s correlation test TGNC: transgender and gender nonconforming; TNF: tumor necrosis factor; CRP: C-reactive protein

Variable		TGNC cortisol	TGNC TNF alpha	TGNC CRP
Stress	Correlation coefficient	0.87	0.92	0.043
	Sig. (2-tailed)	0.004	0.001	0.808
	N	22	22	22

## Discussion

Political, economic, and ideological systems are at the foundation of the varying levels of stigma and prejudice that are directed against gender minorities, which might promote internalized transphobia and stigma, endangering the person's mental health [14]. Using the PSS-10 questionnaire and salivary biomarkers of stress, this study investigated the experiences of gender minorities as a potential link between gender and the development of stress. In this study, we used the PSS (1983) to measure stress as it is the most commonly used measure of general perceived stress and has adequate internal consistency, medium convergent validity with stressful experiences, and good concurrent validity in mental health issues, such as depression and anxiety [15].

The current study's results indicated a high perceived stress among the TGNC group. Similar results were obtained in a study conducted on transgenders by Pondicherry and Cuddalore, where the mean PSS-10 score was 28 (13). Moreover, in the current study, 82.5% (n=33) of TGNC individuals had high perceived stress. This is in line with a study conducted on a sample of 1,093 transgender persons in the United States who exhibited a very high prevalence of clinical depression (44.1%), anxiety (33.2%), and somatization (27.5%) [16].

Since saliva is sensitive, stable, non-invasive, and simple to assess, we decided to use it in our investigation. It is stable for a week at 4 °C and for 24 hours at room temperature without coagulation [17]. Saliva contains elements that reduce HIV's ability to infect, making oral transmission rates exceptionally low and thus making it safer to handle [18]. In the current study, the salivary cortisol levels of the TGNC group had a strong positive correlation with the PSS-10 scores. The lack of anxiety from needle phobia during collection is a clear benefit of salivary assessment against serum cortisol assessment, which could potentially skew the results [19].

In a study conducted by Colizzi et al. [20], persons with gender dysphoria showed a higher cortisol awakening response, higher perceived stress, and increased attachment insecurity than the control population before the start of gender-affirming hormone therapy. This is in accordance with a study conducted among transgenders of Pondicherry and Cuddalore, where a strong positive correlation was seen between the PSS-10 scores and salivary cortisol levels [13]. By contrast, DuBois et al. demonstrated that elevated cortisol was not associated with general perceived stress, but with transitioning-identity stress, frequent "coming-out" stress, and gender-specific public bathroom stress [21]. In the present study, the PSS-10 scores of the TGNC group were positively correlated with salivary TNF-α levels. Clinical depression has been linked to increased TNF-α [22], which has been further proven by a meta-analysis [23]. In a study conducted by Maes et al. [24], students with high stress perception during stressful life events exhibited a noteworthy increase in the secretion of TNF-α.

In the current study, contrary to our expectations, CRP was not significantly correlated with perceived stress. CRP has been discovered to rise in reaction to acute injuries or infections, as well as acute and chronic psychosocial stress, because it is an acute phase protein generated as a response to elevated cytokine production [24]. Previous research has shown that depressed patients have higher levels of CRP [25,26]. However, as opposed to this, a study conducted to assess circulating inflammatory markers in healthy young adults also did not prove CRP to be statistically related to psychological distress, although CRP was correlated with fibrinogen [27].

This study has brought to our attention that not every person who encountered gender incongruence was able to fix it. Future studies should therefore focus on determining what prevents some TGNC individuals from addressing their gender incongruence and psychological discomfort. Future research on a related subject should take into account a diverse transgender community and a larger sample size. In addition, there is a need to create scales that more accurately reflect gender dysphoria and associated stress in the context of an Indian cultural setting. Despite the study's limited sample size, this outcome supports recent World Health Organization advancement to remove transgender health conditions from the Mental and Behavioral Disorders categorization to a fresh chapter on Sexual Disorders and Conditions Related to Sexual Health.

Limitation

The study was done with a modest sample size. As a result, the findings' generalizability is constrained. As the scale was not initially designed for use in an Indian setting, it may be less likely to accurately reflect the complex sensations of dysphoria in an Indian context. Finally, as the measured biomarkers are diurnal, saliva collection should have been carried out at different time points.

## Conclusions

In line with the above reports, it can be inferred that a significant association exists between salivary biomarkers of stress and perceived stress among gender minorities in Chennai, India. Biomarkers for stress can be effective tools for better describing and understanding the health status and health disparities of gender minorities. They can also serve as a powerful instrument for increasing awareness among policymakers so that the policies are more inclusive and are better suited to the individual requirements of gender minorities.
